# Identification and Expression Analysis of Cytochrome P450 Genes Probably Involved in Triterpenoid Saponins Biosynthesis in *Astragalus mongholicus*

**DOI:** 10.3390/ijms25158333

**Published:** 2024-07-30

**Authors:** Junxiu Wang, Baoping Yang, Fusheng Zhang, Jiaorui Wang, Kunlun Xue, Babar Hussain Chang, Jianqin Zhang, Xuemei Qin

**Affiliations:** 1 Modern Research Center for Traditional Chinese Medicine, Shanxi University, Taiyuan 030006, China; wangjx0419@163.com (J.W.); ybp199906@163.com (B.Y.); ample1007@163.com (F.Z.); jr990705@163.com (J.W.); 2 College of Life Science, Shanxi University, Taiyuan 030006, China; 13833410699@163.com (K.X.); babar_chang@yahoo.com (B.H.C.); 3 Faculty of Crop Protection, Sindh Agriculture University, Tandojam 70060, Pakistan

**Keywords:** cytochromes P450 (P450s), expression analysis, astragaloside biosynthesis, *Astragalus mongholicus*

## Abstract

Cytochromes P450 (P450s) are one of the largest enzymatic protein families and play critical roles in the synthesis and metabolism of plant secondary metabolites. Astragaloside IV (AS-IV) is one of the primary active components in *Astragalus* herbs, exhibiting diverse biological activities and pharmacological effects. However, P450s involved in the astragaloside biosynthesis have not been systematically analyzed in *Astragalus mongholicus* (*A. mongholicus*). In this study, we identified 209 P450 genes from the genome of *A. mongholicus* (AmP450s), which were classified into nine clans and 47 families and performed a systematic overview of their physical and chemical properties, phylogeny, gene structures and conserved motifs. Weighted gene co-expression network analysis (WGCNA) revealed that AmP450s are critical in the astragaloside biosynthesis pathway. The expression levels of these AmP450s were verified by quantitative real-time PCR (qRT-PCR) analysis in the root, stem and leaf, showing that most AmP450s are abundant in the root. Additionally, the correlation analysis between gene expressions and AS-IV content showed that twelve AmP450s, especially *CYP71A28*, *CYP71D16* and *CYP72A69*, may have significant potential in the biosynthesis of astragaloside. This study systematically investigates the P450s of *A. mongholicus* and offers valuable insights into further exploring the functions of CYP450s in the astragaloside biosynthesis pathway.

## 1. Introduction

Cytochromes P450 (P450s) are membrane-bound hemoproteins and widely found in living organisms, from bacteria to plants, animals and humans. In plants, P450s, classified into two main clades, A-type and non-A-type, represent one of the largest protein families, accounting for almost 1% of the protein-coding genes [1]. In order to standardize the naming of the P450, the “P450 Nomenclature Committee” made a rule based on the amino acid sequence homology and phylogenetic association of P450s [2]. Specifically, the names of all the cytochrome enzymes consisted of “CYP”, a family number and a subfamily letter, with families and sub-families divided based on the sequence similarity with >40% and >50%, respectively [2,3]. P450s are classified into 10 clans including CYP71, CYP72, CYP85, CYP86 and CYP51, CYP74, CYP97, CYP710, CYP711 and CYP727 clan in plants [4]. The first four clans are comprised of multiple family members and are named after their lowest-numbered family members, while the other six clans contain only one family number [4]. Despite the diversity in their primary amino acid sequence, a few domains in P450s are highly conserved. For example, the proline-rich membrane hinge, the I-helix (AGxD/RT) and the E-R-R triad, which includes the Glu and Arg of the K-helix consensus sequence (KeTLR) and the Arg in the “PERF” consensus sequence [5,6]. Among them, the E-R-R triad, located in the heme-binding domain, is generally regarded as critical for locking the heme pockets into position and conserved in all P450s sequences [5,6]. 

Plant P450s always contribute to biotic and abiotic stress tolerance, including drought, salinity, chemical toxicity, oxidative stress and pest infestation [2,7,8]. In addition, several studies have demonstrated that P450 genes, acting as versatile catalysts, are critical for the synthesis and metabolism of plant secondary metabolites [9,10,11]. The members of the CYP71 clan, which has the largest number, are involved in the biosynthesis of terpenoids, flavonoids, alkaloids, cyanomino acids and fatty acids [2,9,12]. These bioactive natural products are significant in medicine, beauty, agriculture and other fields. Members of the CYP85 clan mainly participated in the biosynthesis of terpenoids [2,9,12], while members of the CYP86 and CYP72 clans were mainly involved in the biosynthesis of terpenoids and fatty acids [2,9,12]. The CYP51 family is involved in modifying the sterol precursors and triterpene [12]. P450s performed diverse catalytic reactions on the various substrates, including oxidation, hydroxylation, epoxidation, dealkylation, C–C cleavage, desaturation, decarboxylation, dimerization, isomerization and ring extension reactions [11,13]. As more genomic data become available, an increasing number of P450s have been identified in plants. For example, 245, 356, 315, 334, 310, 174, 300, 457, 373 and 263 P450s have been reported respectively in *Arabidopsis thaliana* [5], *Oryza sativa* [4], *Vitis vinifera* [14], *Linum usitatissimum* [15], *Populus trichocarpa* [13], *Morus notabilis* [14], *Theobroma cacao* [14], *Solanum lycopersicum* [14], *Gossypium raimondii* [14] and *Zea mays* [14]. In Leguminosae, there are 346 P450s in *Medicago truncatula* [8], 332 in *Glycine max* [16] and 226 in *Cajanus cajan* [17], respectively. However, a comprehensive identification and analysis of P450 members in *Astragalus mongholicus* has not yet been carried out to date. 

Astragali Radix, a traditional Chinese medicine with a long history, is derived from the dry roots of *Astragalus membranaceus* (Fisch.) Bge. var. *mongholicus* (Bge.) Hsiao (*A. mongholicus*) or *Astragalus membranaceus* (Fisch.) Bge (*A. membranaceus*) based on the People’s Republic of China Pharmacopoeia 2020. Astragaloside IV (AS-IV) is one of the primary active ingredients, playing a protective role in cardiovascular disease, pulmonary disease, liver fibrosis, and diabetic nephropathy [18,19]. However, the low contents of AS-IV in plants limits its development and utilization in medicine. Synthetic biology is becoming an effective approach for the synthesizing of natural active ingredients, but the enzymes involved in the biosynthesis of these natural products need to be clearly defined. 

**Figure 1:**
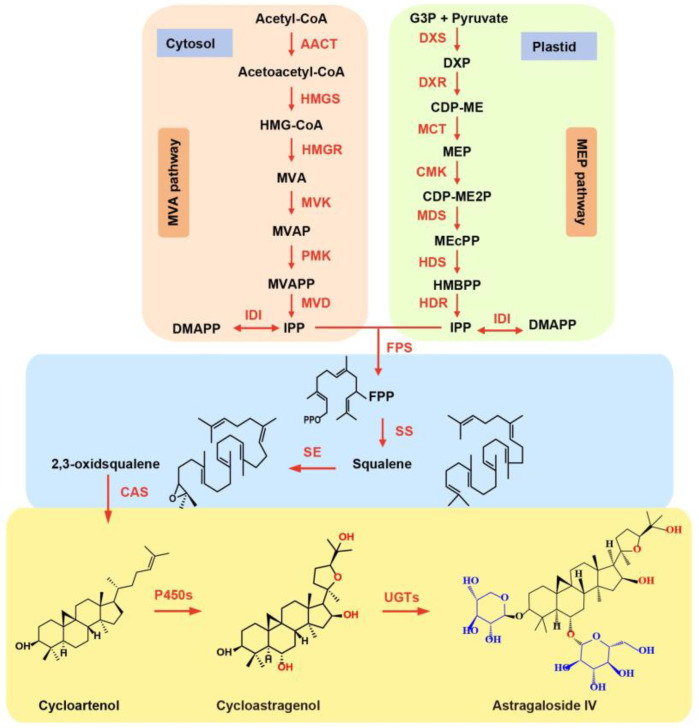
The potential biosynthetic pathways of astragaloside. (AACT, acetoacetyl-CoA thiolase; CMK, 4-(cytidine 5′-diphospho)-2-C-methyl-D-erythritol kinase; DXR, 1-deoxy-D-xylulose 5-phosphate reductoisomerase; DXS, 1-deoxy-D-xylulose 5-phosphate synthase; FDS, farnesyl diphosphate synthase; HDR, (E)-4-hydroxy-3-methylbut-2-enyl diphosphate reductase; HDS, (E)-4-hydroxy-3-methylbut-2-enyl diphosphate synthase; HMGR, 3-hydroxy-3-methylglutaryl-CoA reductase; HMGS, 3-hydroxy-3-methylglutaryl-CoA synthase; IDI, isopentenyl diphosphate isomerase; MCT, 2-Cmethyl-D-erythritol 4-phosphate cytidylyltransferase; MDS, 2-C-methyl-D-erythritol 2,4-cyclodiphosphate synthase; MVD, mevalonate diphosphate decarboxylase; MVK, mevalonate kinase; PMK, phosphomevalonate kinase).

AS-IV is a tetracyclic triterpene, whose precursors metabolic pathway have been identified (Figure 1). Triterpenoids are derived from isopentenyl diphosphate (IPP) and its allylic isomer dimethylallyl diphosphate (DMAPP), which originated from the mevalonate (MVA) pathway and methylerythritol phosphate (MEP) pathway, respectively [20,21]. IPP and DMAPP are condensed to farnesyl diphosphate (FPP) with the assistance of FPP synthase (FPS). 2,3-oxidosqualene, the common precursor of triterpenoids, is transformed from FPP by squalene synthase (SS) and squalene epoxidase (SE) [20]. Subsequently, oxidosqualene cyclases (OSCs) catalyze 2,3-oxidosqualene into various triterpene precursors, such as α- amyrin, β- amyrin, lupeol and cycloartenol. Finally, these triterpene precursors undergo oxidation, hydroxylation, and glycosylation by modifying enzymes, such as P450s and uridine diphosphate glycosyltransferases (UGTs), and eventually generating diverse triterpene saponins [21] (Figure 1). So far, the cycloartenol synthase (CAS) and partial UGTs involved in the biosynthesis of astragaloside have been identified [21,22,23,24]. Although P450s in *A. mongholicus* (AmP450s) have been reported previously [21], P450 genes involved in astragaloside biosynthesis have not been systematically explored in *A. mongholicus.*

In this study, 209 P450 genes were identified from *A. mongholicus* and performed bioinformatics analysis, including physical and chemical properties, evolution, gene structure and conserved motifs. Additionally, the expressions of P450 genes in imitative wild *A. mongholicus* (WAM) and cultivated *A. mongholicus* (CAM) were analyzed using weighted gene co-expression network analysis (WGCNA) and identified some candidate P450s involved in astragaloside biosynthesis, which was verified by quantitative real-time PCR analysis (qRT-PCR) in different tissues, including root, stem and leaf. Finally, the content of AS-IV in *A. mongholicus* seedlings was measured, and its correlation with the expressions of AmP450 genes was analyzed. These results present a comprehensive overview of P450 genes in *A. mongholicus*, including their phylogenetic analysis, expression profiles characterization and the relationship with astragaloside biosynthesis, offering valuable insights into further exploring the functions of P450s in the astragaloside biosynthesis pathway. 

## 2. Results

### 2.1. Identification of P450 Genes in A. mongholicus

In this study, a total of 243 AmP450s were obtained from the Global Pharmacopoeia Genome Database (GPGD), and 34 of them were considered as allelic variants due to their high sequence similarity (>97%), consequently. Ultimately, 209 full-length AmP450 genes, encoding proteins ranging from 349 (CYP704B1) to 940 (CYP736A13) amino acids, were retained for further analyses (Table S1). Amino acid sequence prediction analysis shows that the molecular weight of AmP450 proteins ranges from 39.82 to 106.79 kDa. The theoretical isoelectric points of AmP450s span from 8.68 to 8.85. Subcellular localization analysis indicates that the vast majority of AmP450s are positioned in the endoplasmic reticulum (ER), except for CYP74A1, CYP74A2 and CYP701A16, which are located in chloroplast (Table S1).

For official nomenclature, AmP450s were named based on the amino acid sequence similarity and phylogeny compared to *Arabidopsis* (Figure S1). A total of 209 AmP450s were divided into 9 clans and 47 families, including CYP51, CYP71, CYP72, CYP74, CYP85, CYP86, CYP97, CYP711 and CYP727 clans. The CYP71, CYP72, CYP85 and CYP86 clans comprise multiple families, including 18, 3, 8 and 3 families, respectively. The CYP51, CYP74, CYP97, CYP711 and CYP727 clans each contain only one family (Table S1).

**Figure 2:**
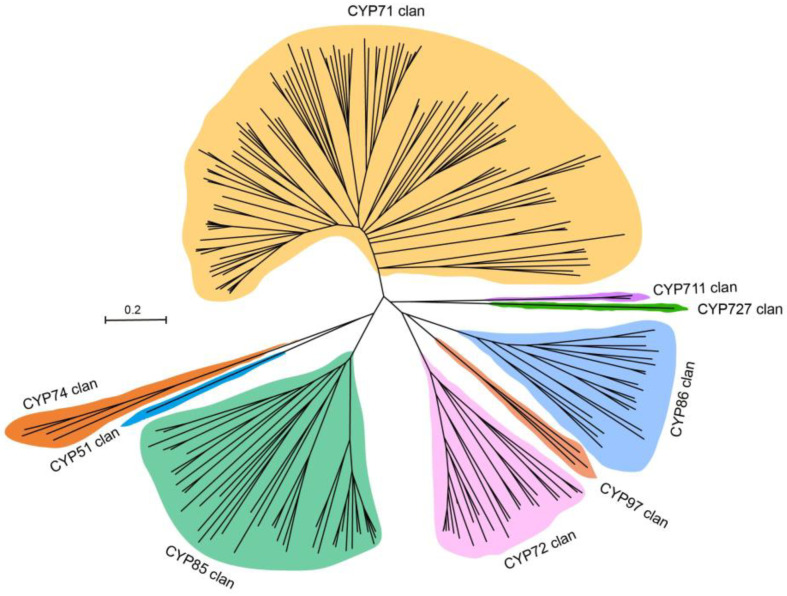
Phylogenetic tree of P450 gene family of *A. mongholicus*. Different colors represent different clusters.

To understand the phylogenetic relationships of AmP450s, a phylogenetic tree was generated from the 209 AmP450s protein sequences using MEGA-X. The phylogenetic tree showed that AmP450s are grouped into three clusters. (Figure 2). The first cluster contains only the CYP71 clan, which accounts for more than 53% of the P450s (111 out of 209) in *A. mongholicus*. The second cluster consists of CYP51, CYP74 and CYP85 clans, including 1, 4 and 40 genes, respectively. Additionally, the CYP72, CYP97, CYP86, CYP711 and CYP727 clans are clustered together, including 25, 3, 21, 2 and 1 genes, respectively (Figure 2, Figure S2 and Table S1).

### 2.2. Gene Structure and Conserved Motifs Analysis of AmP450s

**Figure 3:**
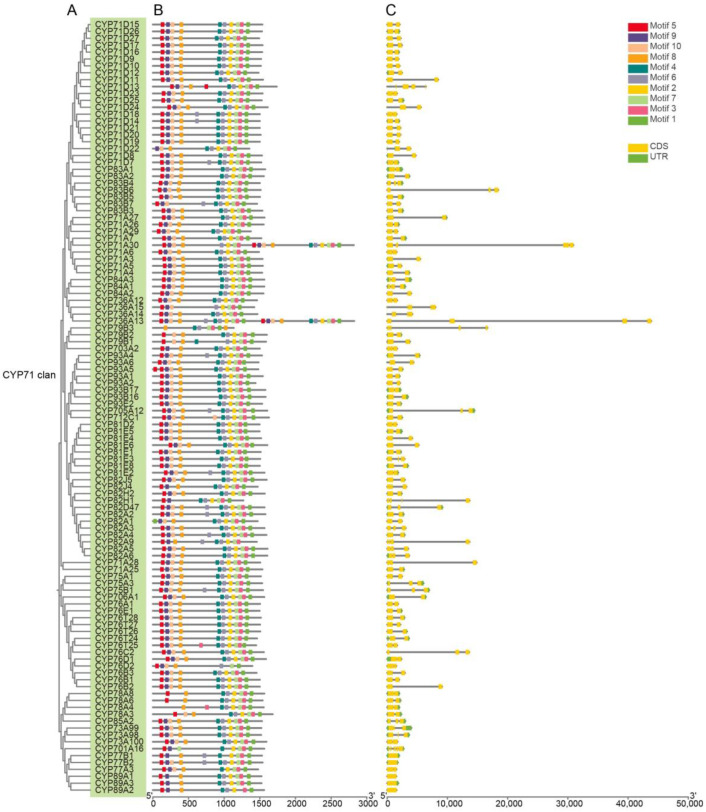

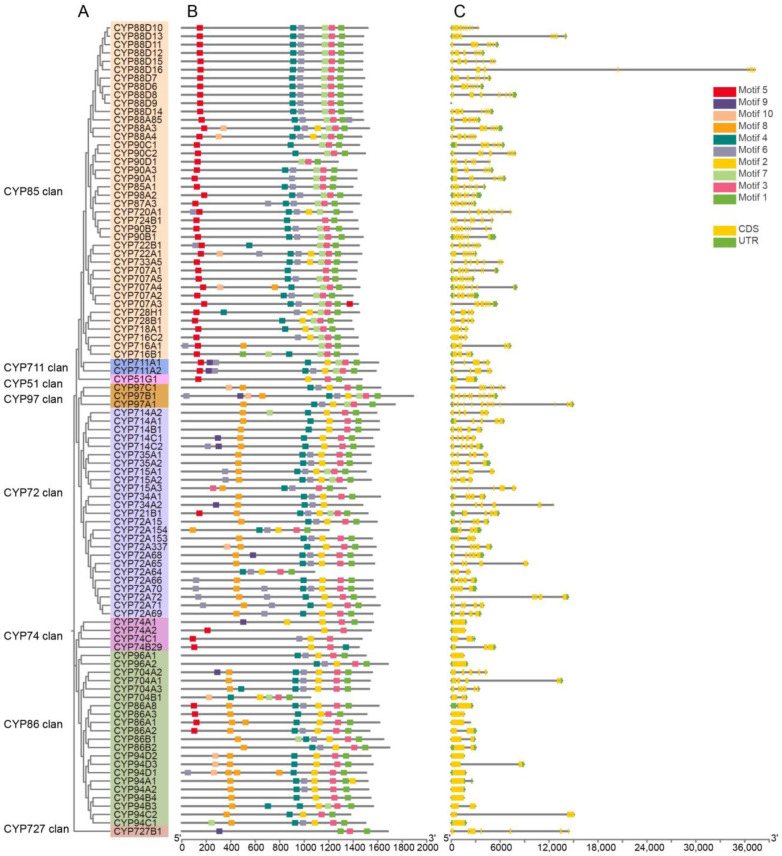
Conserved motifs and gene structures of P450s from *A. mongholicus*. (**A**) Phylogenetic relationship; (**B**) conserved motifs; (**C**) gene structures.

To gain sights into the structural features of AmP450 genes, the number of exons and introns was analyzed based on the *A. mongholicus* genome. Within the CYP71 clan, most of the members have 2–4 exons except for CYP736A13, CYP82A9 and CYP736A15, which have five, five and six exons. Additionally, CYP701A16 and CYP79B3 have seven exons (Figure 3A,C). In the CYP72 clan, most genes have five exons, except for CYP72A64, CYP72A154 and CYP734A3, which have three, four and four exons, respectively. Three members of CYP97 clan have over eight exons, with CYP97C1 having nine exons, CYP97B1 having 15 exons (the maximum exon number of 209 AmP450s), followed by CYP97A1 having 14 exons. In CYP86, CYP74, CYP51, and CYP711 clans, most of the members have 2 to 6 exons, while 8 of the 21 CYP86 clan genes and 2 out of the 4 CYP74 clan genes have a single exon. Most members of the CYP85 clan have over eight or nine exons, while four members of the CYP716 family (CYP718A1, CYP716C2, CYP716A1 and CYP716B1) have three or four exons (Figure 3C).

For a general view of the function of AmP450 proteins, 10 conserved motifs were identified using MEME (Figure 3B and Figure S3). Notably, motifs such as the FXXGXRXCXG motif (motif 2) in the heme-binding domain, the EXXR motif (motif 1) in the K-helix, the AGxDT motif (motif 8) in the I-helix and PXRX motif (motif 3) are conserved motifs across P450 proteins. Additionally, the motifs 6 and 4 in the C-terminal region of most AmP450s are conserved. However, some variations exist among them. Specifically, most CYP71 clan proteins contain 10 motifs, except for 17 of them missing motifs 5, 8 or 9. The CYP72 clan proteins contain 5–8 motifs except for CYP72A70, with eight motifs. The CYP74 clan proteins typically have 3–5 motifs. Members of the CYP97 clan lack motifs 5, 6, 9 or 10, containing 7–9 motifs. In the CYP86 clan, proteins contain 5–8 motifs and lack motifs 5, 9 or 10, except for CYP86D1, which has five motifs. The CYP51G1 contains only five motifs. The CYP711 and CYP85 clans contain six or nine motifs, missing motifs 2 and 8–10 (Figure 3B). Overall, most AmP450 proteins within the same clan possess similar motifs, suggesting potential functional similarities among these AmP450 members.

### 2.3. Analysis of the AmP450 Genes Involved in Astragaloside Biosynthesis by WGCNA

**Figure 4:**
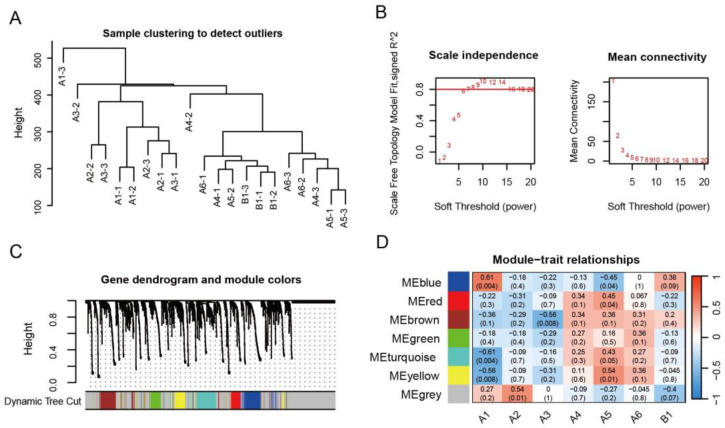
Weighted gene co-expression network analysis among P450 genes associated with the different growth stages of *A. mongholicus.* (**A**) Dendrogram of all 21 samples; (**B**) soft threshold calculation of gene co-expression network; (**C**) module detection by gene cluster dendrogram. (**D**) module-trait associations revealed by Pearson correlation coefficient. The left color column represents different co-expression modules. The right color scale from −1 (blue) to 1 (red) presents module trait correlations. The numbers in the Figure indicate the module-trait correlation and corresponding *p*-value.

WGCNA is a systematic biological method used to calculate gene networks involved in related traits rather than individual genes. To gain insights into the AmP450 genes involved in the astragaloside biosynthesis, WGCNA was performed using fragments per kilobase of transcript per million mapped reads (fpkm) value of all AmP450s from the transcriptome data of 24 samples, which contains 1 to 6-year-old WAM (A1-A6) and 1 to 2-year-old CAM (B1-B2). The analysis included triterpene precursor biosynthesis genes, such as *HMGR*, *DXR*, *MVK*, *MVD*, *HDS*, *HDR*, *MCT*, *CMK*, *FPS*, *SS*, *SE* and *CAS* (Table S2). Initially, we analyzed the correlation coefficients of expression level for each sample by clustering 24 samples (Figure S4) and removing the three outlier samples (B2-1, B2-2 and B2-3). The clustering tree composed of 21 samples is shown in Figure 4A. Based on a scale-free topology fit index of 0.8, we defined 10 as the soft threshold (Figure 4B). Gene clustering was performed based on TOM-based dissimilarity, and modules were identified using a dynamic tree cut. The results revealed seven co-expression modules (Figure 4C), with the blue module containing the largest number of genes (34), second only to the gray module (57), and the pink module comprising the fewest genes with 22. As the content of AS-IV is higher in the 1-year-old WAM [25], we focused on modules related to the early development stage of *A. mongholicus* as candidate genes. The modules-trait relationship indicated that the blue module was closely associated with 1-year-old WAM (r^2^ = 0.61, *p* = 0.004) (Figure 4D). Additionally, the grey module contained five astragaloside biosynthesis genes (*HMGR*, *CMK*, *SS*, *SE* and *CAS*) and showed a significant correlation with A2 of WAM (r^2^ = 0.54, *p* = 0.01) (Figure 4D). Thus, the blue and grey modules were selected as the modules of interest.

**Figure 5:**
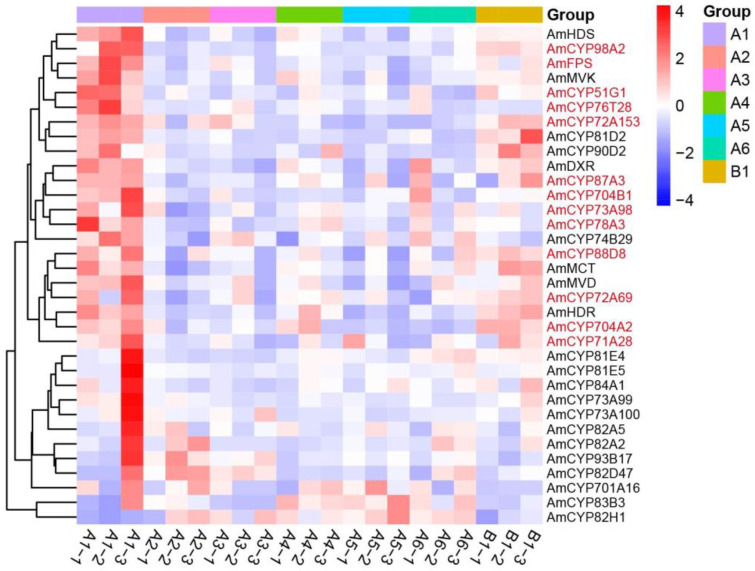
Heat map of AmP450 gene expressions from the blue module. A1–A6, 1–6-year-old WAM; B1, 2-year-old CAM. Each sample contains three biological replicates.

**Figure 6:**
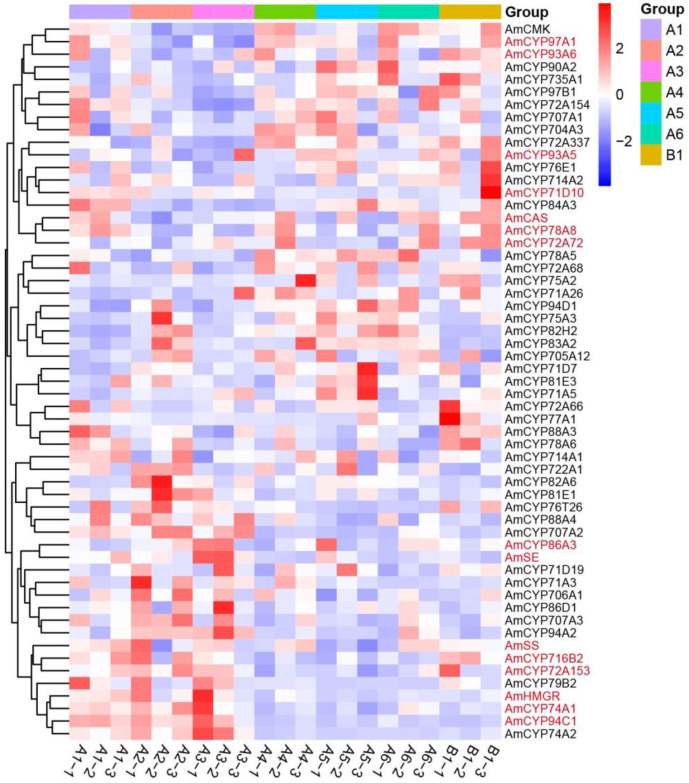
Heat map of AmP450 gene expressions from the grey module. A1–A6, 1–6-year-old WAM; B1, 2-year-old CAM. Each sample contains three biological replicates.

To further elucidate the gene expressions and correlations within the blue and grey modules, heat maps were generated using the fpkm value (Figure 5 and Figure 6). The results showed that the *FPS*, *MVK*, *MVD*, *HDS*, *HDX* and *DXR*, six biosynthesis genes of triterpene precursors, are distributed in the blue module. Among them, 14 AmP450s, including *CYP98A2*, *CYP51G1*, *CYP87A3* and *CYP72A69,* showed similar expression patterns and were highly expressed in the 1-year-old WAM (A1-1, A1-2 and A1-3) of *A. mongholicus* (Figure 5). Similarly, 15 AmP450s exhibited similar expression patterns to *HMGR*, *CMK*, *SS*, *SE*, and *CAS*, which were located in the grey module (Figure 6). These findings suggest that the AmP450 genes in the blue and grey modules may play crucial roles in astragaloside biosynthesis.

### 2.4. Expression Profiles of AmP450 Genes in Different Tissues of A. mongholicus

**Figure 7:**
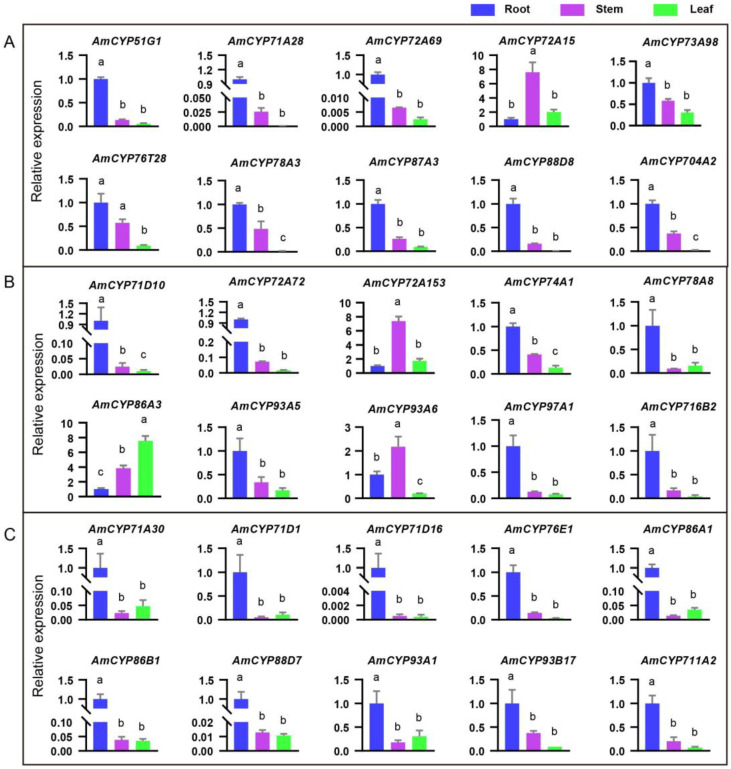
Relative expressions of the AmP450 genes in the root, stem and leaf of 4-week-old *A. mongholicus* seedlings by qRT-PCR. (**A**) Blue module; (**B**) Grey module; (**C**) Other genes. The results are presented as the means ± standard error (*n* = 3). Different letters indicate significant differences at *p* < 0.05, determined by Tukey’s test of one-way analysis of variance (ANOVA).

To further understand the roles of *AmP450* genes in *A. mongholicus,* 13 and 15 AmP450 genes from blue and grey modules were selected for qRT-PCR analysis using the tissues of *A. mongholicus* seedling, including root, stem and leaf. Most genes are highly expressed in the root except for *CYP93A6*, *CYP72A15*, *CYP704B1* and *CYP72A153*, which are highly expressed in the stem and *CYP86A3*, which is highly expressed in the leaf (Figure 7A,B and Figure S5). Notably, *CYP71A28*, *CYP71D16*, *CYP72A69*, *CYP71D10* and *CYP72A72* transcripts are 2048-fold, 2357-fold, 405-fold, 92-fold and 60-fold more abundant in the root than those in the leaf, and 39-fold, 1837-fold, 153-fold, 40-fold and 13-fold more abundant in the root than them in the stem. Additionally, *CYP94C1* is widely expressed in the root, stem and leaf. The expressions of *HMGR*, *FPS*, *SS*, *SE* and *CAS* are detected in three tissues but are still highest in the root (Figure S5). This indicated that the synthesis of triterpenoid precursors occurs in various tissues.

Furthermore, to understand broader expression characteristics of P450 genes, an additional 18 genes were examined, including *CYP93B17*, *CYP71D16*, *CYP76E1* and others. These results showed that a total of 13 AmP450 genes were highly expressed in the root compared to the leaf and stem (Figure 7C and Figure S5). Among them, *CYP71A30*, *CYP71D16*, *CYP86A1*, *CYP86B1* and *CYP88D7* transcripts display significant tissue specificity in the root. Especially *CYP71D16*, whose transcripts are 1837-fold more abundant in the root than in the stem and 2357 more abundant in the leaf. Additionally, there were 3 AmP450 genes (*CYP86A3*, *CYP97C1* and *CYP714D14*) that were highly expressed in the stem and leaf. Collectively, these results highlight the tissue-specific expression patterns of AmP450 genes, which indicated that AmP450s may be involved in the growth and development of *Astragalus* plants as well as the synthesis of secondary metabolites.

### 2.5. A Closely Relationship between Expression of P450s and AS-IV Biosynthesis

**Figure 8:**
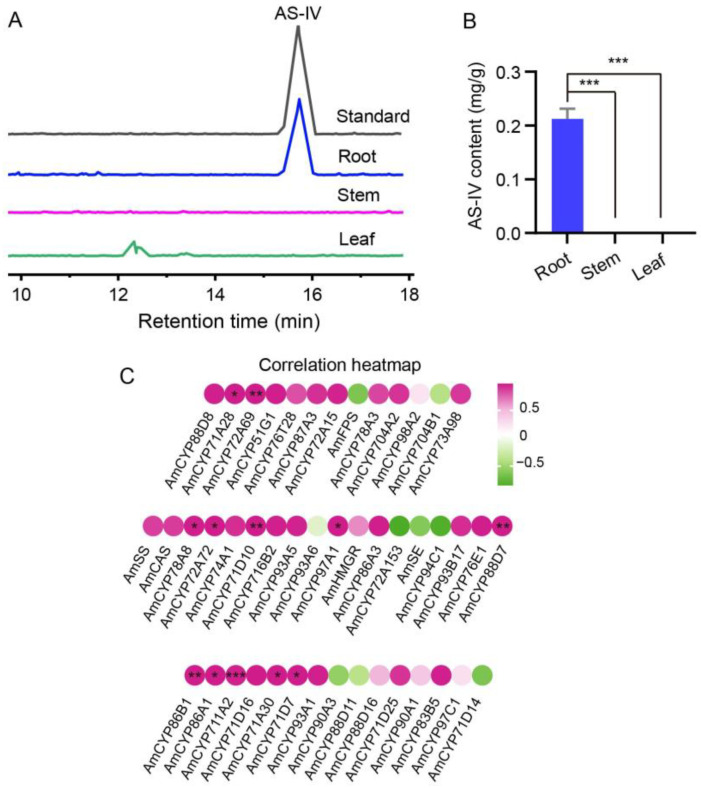
Correlation analysis between AS-IV content and mRNA expression level of astragaloside biosynthesis genes. (**A**) HPLC-ELSD analysis of AS-IV content in the root, stem and leaf of 4-week-old *A. mongholicus* seedlings. (**B**) The content of AS-IV in the root, stem and leaf of *A. mongholicus* seedlings. (**C**) Pearson correlation analysis between AS-IV content and mRNA expression level of astragaloside synthesis genes of *A. mongholicus.* Statistical significance was analyzed using Student’s *t*-test (* *p* < 0.05, ** *p* < 0.01, *** *p* < 0.001).

To further elucidate the relationship between the expression of AmP450s and the distribution of AS-IV in *Astragalus* seedlings, the AS-IV contents in the root, stem and leaf of 4-week-old seedlings were detected by high-performance liquid chromatography with evaporative light scattering detector (HPLC-ELSD) method. The results showed that the AS-IV was predominantly present in the root, with a concentration of up to 0.2mg/g, while it was almost undetectable in the stem and leaf (Figure 8A,B). This result suggests that the root is likely the primary site of astragaloside synthesis.

Subsequently, Pearson correlation analysis between AmP450 gene expression and the AS-IV content was further conducted. The results revealed that most AmP450s were positively correlated with AS-IV content. Notably, 12 AmP450s showed a significant correlation with AS-IV level including *CYP71A28*, *CYP71A30*, *CYP71D7*, *CYP71D10*, *CYP71D16*, *CYP72A69*, *CYP72A72*, *CYP78A8*, *CYP86B1*, *CYP86A1*, *CYP88D7* and *CYP97A1* (Figure 8C). These findings indicate that these AmP450 genes have substantial potential as candidate genes involved in the biosynthesis of astragaloside.

## 3. Discussion

### 3.1. Comprehensive Identification of P450 Genes in A. mongholicus

The cytochrome P450 gene family is one of the largest families in plants. The amount of P450s varies significantly across different plant species. For example, in the Legume family, *Medicago truncatula*, *Glycine max* and *Cajanus cajan* possess 346, 332 and 226 P450s, respectively [8,16,17]. In the Poaceae family, the P450s number is 1285, 263 and 255 in wheat, maize and rice, respectively [26,27]. In this study, 209 full-length P450 genes were identified from *A. mongholicus*, which is a relatively lower number compared to other plants. The 209 AmP450s were classified into two clades: the non-A type and the A-type. The A-type contains only the CYP71 clan, which is comprised of approximately 53% P450s members in *A. mongholicus*. However, in *M. truncatula* and *Arabidopsis*, the A type P450 genes account for more than 60% (209 out of 346) and 63% (167 out of 264), respectively [8]. This phenomenon may be attributed to the long-term growth of wild *A. mongholicus* in extreme environments, leading to the loss of some functionally redundant genes to conserve energy and enhance survival. Actually, genes involved in the rejection of self-pollen, defense response and negative regulation of molecular function were contracted in the *A. mongholicus* genome [21]. In the non-A type, the CYP72, CYP97, CYP86, CYP711 and CYP727 clans clustered together, while the CYP51, CYP74 and CYP85 clans grouped together, suggesting functional similarities among these clustered AmP450s, which resulted in similar to that reported by Chen et al., 2015 [28]. Surprisingly, CYP710, which is conserved in land plants [13], was not found in *A. mongholicus,* possibly lost during evolution. Both CYP51G and CYP710 members are involved in the biosynthesis of membrane sterols [12], suggesting that AmCYP450s have undergone purifying selection [21], maybe resulting in the retention of CYP51 and the loss of CYP710. Additionally, the CYP727 family was discovered from *A. mongholicus* in this study. It is similar to CYP727A4 (Protein ID: ACG25516.1), with 41.28% identity in maize, and CYP727B3, with 55.53% identity in *Citrus x paradisi*, which are monocots. Furthermore, AmCYP727B1 showed 76.61% similarity to CYP727B5 (https://drnelson.uthsc.edu/plants/) in *Cajanus cajan*, another Leguminosae species. And many homologous genes of AmCYP727B1 were widely found in dicotyledon by blastp in NCBI BLAST, such as 75.80% similarity with *Trifolium pratense* XP_045820783.1), 57.43% similarity with *Camellia lanceoleosa* (KAI7981253.1), 57.7% similarity with *Populus trichocarpal* (XP_024453627.2), indicating that CYP727 family exists in different plant species. Furthermore, CYP82H, a new subfamily, was identified in the CYP82 family, which does not exist in *Arabidopsis* and *Cajanus cajan*. In *A. mongholicus*, there are 25 paralogous gene pairs and 78 tandemly duplicated genes among the AmP450s [21]; therefore, it is speculated that CYP82H may have differentiated into a new subfamily through the amplification or reduction of the other gene families.

### 3.2. AmP450s Involved in Astragaloside Synthesis

P450s have diverse functions not only in the growth and development of plants but also in natural product biosynthesis. AS-IV, one of the primary compounds in *A. mongholicus*, exhibits various pharmacological properties, such as anti-viral, anti-inflammatory, immunomodulatory, antiapoptotic and anti-tumor [18]. However, P450s involved in the astragaloside biosynthesis remain unclear. Therefore, this study aimed to summarize P450 genes in *A. mongholicus* to facilitate the analysis of the astragaloside biosynthesis pathway. The WGCNA and qRT-PCR were used to identify potential AmP450s involved in the astragaloside biosynthesis. The results showed that over 80% of AmP450 genes are highly expressed in the root. However, the correlation analysis between the expression of AmP450s and astragaloside content revealed that 12 AmP450s were strongly correlation with astragaloside synthesis. Among them, 50% of AmP450s are members of CYP71A, CYP71D and CYP78. The remaining AmP450s are distributed in the CYP72 (2), CYP86 (2), CYP88 (1) and CYP97 (1) families, respectively. This result is consistent with the report of Chen et al., which speculated that 22 P450 transcripts belong to CYP71, 72 and 85 clans presumably involved in the biosynthesis of astragalosides through co-expression analysis with CAS [28]. Previous studies have shown that P450s are critical in the biosynthesis of terpenoids. For instance, In *Marrubium vulgare*, CYP71AU87 plays a hydroxylation role in the biosynthesis of marrubiin and related diterpenoids [29]. In *Zingiber zerumbet*, CYP71BA1 also performs hydroxylation in the biosynthesis of zerumbone, a sesquiterpenoid with multiple potential anticancer properties [30]. CYP71AM1 participates in the biosynthetic pathway of the allelochemical sorgoleone [31]. CYP72A67 and CYP72A68 play an oxidative role in the biosynthesis of oleanate sapogenins and hemolytic saponin in *Medicago truncatula* [32]. CYP88L7 and CYP88L8 perform hydroxylation in cucurbitacin biosynthesis of *Momordica charantia* [33]. The member of the CYP97 family is involved in xanthophyll’s synthesis [27]. The above literature research indicated that CYP71, CYP72, CYP85, CYP86 and CYP97 are important for terpenoid synthesis, which indirectly reflects the role of AmP450s in saponin synthesis.

Furthermore, P450s play a vital role in stress response, such as drought, cold, salinization, and herbicide. For example, CYP71D8L regulates rice growth and stress responses by maintaining phytohormone homeostasis [34]. CYP86A2 is involved in the biosynthesis of epicuticular lipids in *Arabidopsis*; a lack of CYP86A2 leads to a reduction in cuticle membrane thickness and increased water permeability, which enhances drought stress response [35]. Under heat and cold stress, the *CYP73A*, *CYP75A* and *CYP75B* are significantly upregulated in *Lolium perenne* and *Festuca arundinacea* [36]. CYP81D5 enhances salinity tolerance in wheat by accelerating reactive oxygen scavenging activity [37]. CYP88A1 involved in gibberellin biosynthesis, is highly expressed in aluminum-stressed wheat roots [38]. Some P450 genes are involved in herbicide metabolism, such as *CYP81A6* in rice and CYP71C6v1 in wheat [39]. These studies suggested that P450s are critical for improving stress-tolerant plants.

In our study, the expression of *CYP72A15*, *CYP93A6*, *CYP704B1*, *CYP72A153*, *CYP88D11* and *CYP71D14* preferentially expressed in the stem, and *CYP86A3*, *CYP90A3* are abundant in the leaf of 4-week-old seedlings. Among them, six AmP450 genes contain six motifs except for *CYP93A7* and *CYP71D14*, which include ten motifs, indicating these genes may regulate diverse progress in the growth and development of plants. For instance, members of the CYP86A subfamily and CYP704B1 exhibit fatty acid hydroxylase activities and participate in the synthesis of epidermal cutin and sporopollenin, respectively, in *Arabidopsis* [40]. CYP93A1 and CYP93A2 play a dual role in the isoflavonoid pathway of soybean [41]. CYP90 family regulates the brassinosteroids biosynthesis for the maintenance of plant architecture in rice [42].

In addition, we wondered if the distribution of AS-IV in *Astragalus* seedlings, 4-week-old seedlings, coinciding with the time period used for qRT-PCR, was used to detect the content of AS-IV. The results showed that AS-IV is present only in the root (Figure 8A,B). These results are consistent with the findings of Chen, it is reported that astragaloside (I/II/III/IV) specifically existed in the underground parts of 2 weeks seedlings in *A. membranaceus* [22]. Additionally, we found that the terpenoid synthetic genes, including *FPS*, *SS*, *SE* and *CAS*, were widely expressed in all three tissues (Figure S5), suggesting that cycloartenol, the skeleton of astragaloside, can be synthesized in these tissues. Indeed, cycloartenol exists in the aerial parts [22], which indicates that *Astragalus* saponins are synthesized in the root and hindered in the stem and leaf due to the lack of P450s and UGTs, at least during the seedling stage. This suggests that AmP450, specifically expressed in the root, may play a significant role in astragaloside biosynthesis. For example, the expression of *CYP71D16* in the root is 1837 and 2357 folds higher than in the stem and leaf, respectively. Followed by *CYP71A28* (39, 2048 folds) and *CYP72A69* (153, 405 folds) (Figure 7). In future work, we aim to further verify the function of these AmP450s through heterologous expression in yeast and in plants using hairy roots or callus systems.

## 4. Materials and Methods

### 4.1. Plant Materials

Seeds of *A. mongholicus* were obtained from from Hunyuan County, Datong City, Shanxi Province. Before planting, the seeds were treated with boiling water for 1 min, followed by warm water for 20 min and then covered with gauze for 10 h. The seeds were cultivated in nutrient soil containing 50% vermiculite under 25 °C and 40% relative humidity with a photoperiod of 16 h light/8 h dark. Germination typically occurs 2–3 days after planting, which was recorded as the first day of the growth cycle. The 4-week-old seedlings were used for qRT-PCR and HPLC analysis.

### 4.2. Identification of CYP450 Genes from A. mongholicus

A total of 243 AmP450 sequences were obtained from the GPGD (http://www.gpgenome.com/species/109, accessed on 10 January 2024), and further submitted to BLAST analysis against plant P450s. According to the rule of CYP450 International Nomenclature Commission (http://drnelson.uthsc.edu/CytochromeP450.html, accessed on 25 May 2024), the amino acid sequences exhibiting >40%, >50% and >97% similarity were assigned to the same family, subfamily and allelic variants respectively. 34 allelic variants of AmP450s were excluded with the similarity of proteins sequence >97%, and 209 full length AmP450 genes was identified.

### 4.3. Sequence Analyses, Structural Characterization and Phylogenetic Tree Construction

The physicochemical properties of AmP450s proteins, including the number of amino acids, molecular weight and theoretical isoelectric points of proteins, were calculated by the online Expasy tool (Expasy—Compute pI/Mw tool). The subcellular localization of AmP450s proteins was analyzed by Plant-mPLoc online tool (http://www.csbio.sjtu.edu.cn/bioinf/plant-multi/, accessed on 8 June 2024). The conserved motifs of AmP450s proteins were identified using the MEME program (MEME-Suite version 5.5.5, MEME—Submission form (meme-suite.org)) [43]. The motif number was set to 10, and the width of the motif was minimum = 10, maximum = 50. Conserved motifs and gene structures of AmP450s were shown using TBtool [44]. AmP450s sequences were aligned by clustaIW with the default setting. Phylogenetic trees were generated based on the amino acid sequence using MEGA-X software (version 10.2.6) with the maximum likelihood estimation (MLE) method and default parameters. P450 sequences of *Arabidopsis thaliana* were downloaded from Plants Cytochrome P450 (https://drnelson.uthsc.edu/plants/, accessed on 13 June 2024). Phylogenetic trees are displayed by the iTOL online tool (https://itol.embl.de/, version 6.9.1) [45].

### 4.4. Weighted Gene Co-Expression Network Analysis

The co-expression gene network of all AmP450 genes from different growth years of *A. mongholicus* was constructed using the WGCNA package [46]. Briefly, the fpkm of AmP450 genes were obtained from transcriptome databases of different growth years of *A. mongholicus* [47], whose sequences were compared with the genome database (Table S3). These data were loaded to data input packages of R and checked for missing values. The samples were clustered by average value to determine if there are outliers, and visualized sample dendrogram. Second, the network constructions and module detections were performed with the following parameters: ablineHeigh: 0.8, softPower: 10, minModuleSize: 40, and all genes were grouped into different modules. Relationships between traits and modules were identified by Pearson correlation analysis. The fpkm values of AmP450s are shown in Table S2. Data analysis and visualization of heat maps were graphed by a free web server named SRplot (https://www.bioinformatics.com.cn, accessed on 15 May 2024) [48].

### 4.5. Gene Expression Analysis by qRT-PCR

To compare the mRNA expression levels of the AmP450s in different tissues, qRT-PCR was performed using the Bio-Rad instrument (Bio-Rad, Hercules, CA, USA). Total RNA was isolated from the leaf, root and stem of 4-week-old *A. mongholicus* seedlings using an RNAprep Pure Plant kit (#DP432, Tiangen, Beijing, China). Three independent biological replicates were prepared, and each replicate contained five samples. Then, 1 μL total RNA was reverse-transcribed using reagents and enzymes from Takara Bio Group (Otsu, Shiga, Japan). The reaction volume of qRT-PCR was 20 μL including 10 μL of 2×StarLighter SYBR Green qPCR Mix (Forever Star, Beijing, China), 0.75 μL of forward and reverse primers (10 μM), 4 μL of 20 fold diluted cDNA and 4.5 μL of ddH_2_O. PCR amplification was conducted at 95 °C for 30 s, followed by 95 °C for 5 s, 52 °C for 30 s, and 72 °C for 30 s with 40 cycles. The melting curves were added at 65 °C. 18S RNA was used as the reference gene. Relative gene expressions were analyzed using the 2^−ΔΔCT^ methods [49]. The images were completed by GraphPad Prism 8. Primer sequences used in this study are listed in Table S4.

### 4.6. HPLC-ELSD Analysis of the Content of AS-IV

The tissues of root, stem and leaf of 4-week-old *A. mongholicus* seedlings were separately collected and used for the determination of AS-IV content. According to the method of the People’s Republic of China Pharmacopoeia (2020), AS-IV was extracted by condensation and reflux with 80% methanol solution containing 4% concentrated ammonia solution at 70 °C. The filtrate was dried at 70 °C, redissolved in 80% methanol, then transferred to a 5 mL volumetric flask filled with 80% methanol and shaken well. The extracts were determined by HPLC-ELSD (Agilent, Palo Alto, CA, USA) system with Agilent 5 HC-C18 C18 column (4. 6 mm × 150 mm, 5 μm), which was performed as follows: The mobile phase was methanol and water (80:20) flow rate of 1 mL/min. The standard substance AS-IV (Herbest, Baoji, China) was precisely weighed and dissolved in 80% methanol to produce 0.3 and 0.5 mg/mL. AS-IV was quantified using the external standard two-point method. Pearson correlation (*p* < 0.05) was used to analyze the correlation between gene expression and triterpenoid accumulation using the OmicShare tools (https://www.omicshare.com/tools, accessed on 10 June 2024).

### 4.7. Statistical Analysis

One-way analysis of variance (ANOVA) and Tukey’s test of SPSS 16.0 software were used for correlation analysis of gene expression in different tissues. Different letters represent significant differences (*p* < 0.05). The content of AS-IV was evaluated by Student’s *t*-test. Significant differences are marked with asterisks (*** *p* < 0.001). All data are presented as means ± SE. The graphs were completed using GraphPad Prism 8.

## 5. Conclusions

In this study, we concluded a systematic analysis of P450s in *A. mongholicus*, including protein characterization, phylogeny, gene structure, conserved motifs and gene expression profiles, which provide an important basis for gene function analysis. In short, a total of 209 AmP450 genes were identified from *A. mongholicus* and divided into nine clans and 47 families. Conserved motifs analysis showed that all AmP450s contain the signature motifs “FXXGXRXCXG”, “EXXR”, and “PXRX” motifs, which are essential for locking the heme pockets into the heme-binding domain. These motifs possess specific features which may contribute to different molecular functions. Expression analysis based on WGCNA and qRT-PCR data revealed that a majority of AmP450s are abundantly expressed in the root, which is considered the primary storage site for natural active ingredients. Correlation analysis further showed that 12 AmP450s were closely related to AS-IV content, suggesting their critical roles in the biosynthesis of astragaloside. This study provides valuable insights into the functional analysis of P450s in the astragaloside biosynthesis pathway.

## Data Availability

Data are contained within the article or Supplementary Materials.

## Supplementary Materials

The following supporting information can be downloaded at https://www.mdpi.com/article/10.3390/ijms25158333/s1.
