# Supplementary material for: Identification and Expression Analysis of Cytochrome P450 Genes Probably Involved in Triterpenoid Saponins Biosynthesis in Astragalus mongholicus
Source: Int J Mol Sci. 2024 Jul 30;25(15):8333. doi: 10.3390/ijms25158333 (PMC11312233; doi:10.3390/ijms25158333)

Supplmental informations

Figure S1. Phylogenetic tree of P450 gene family in *Arabidopsis thaliana* and *A. mongholicus*.

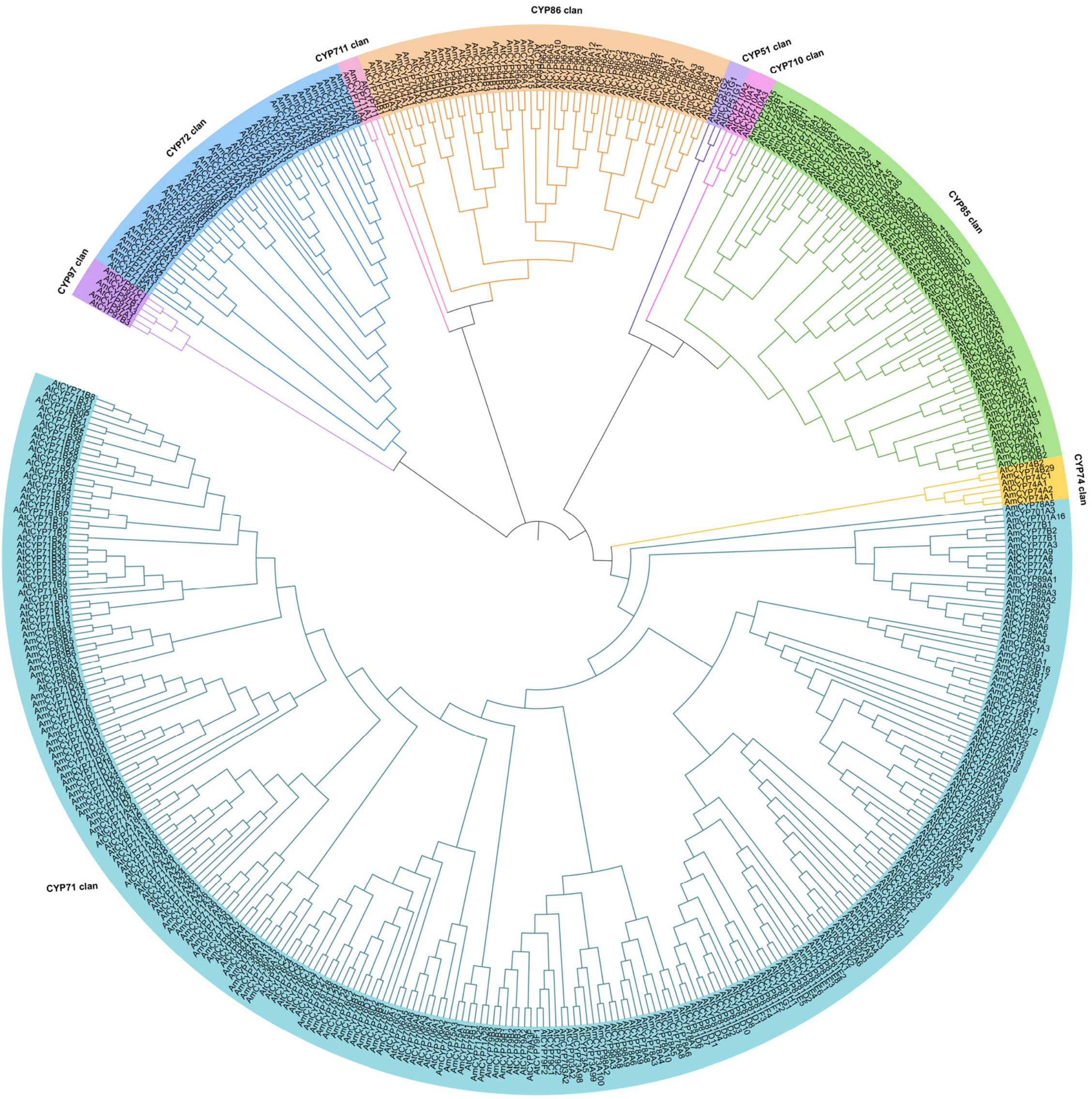

Figure S2. Phylogenetic tree of P450 gene family of *A. mongholicus*.

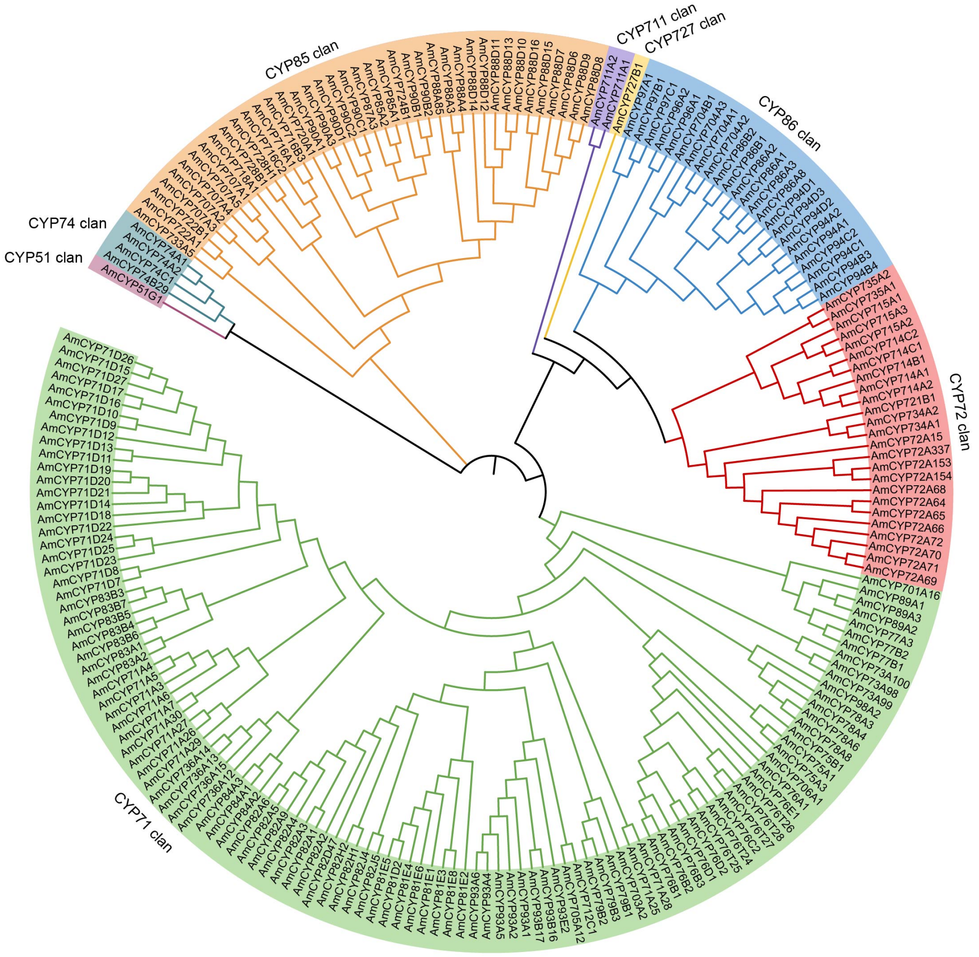

Figure S3. Conserved motifs of CYP450 proteins from *A. mongholicus*.

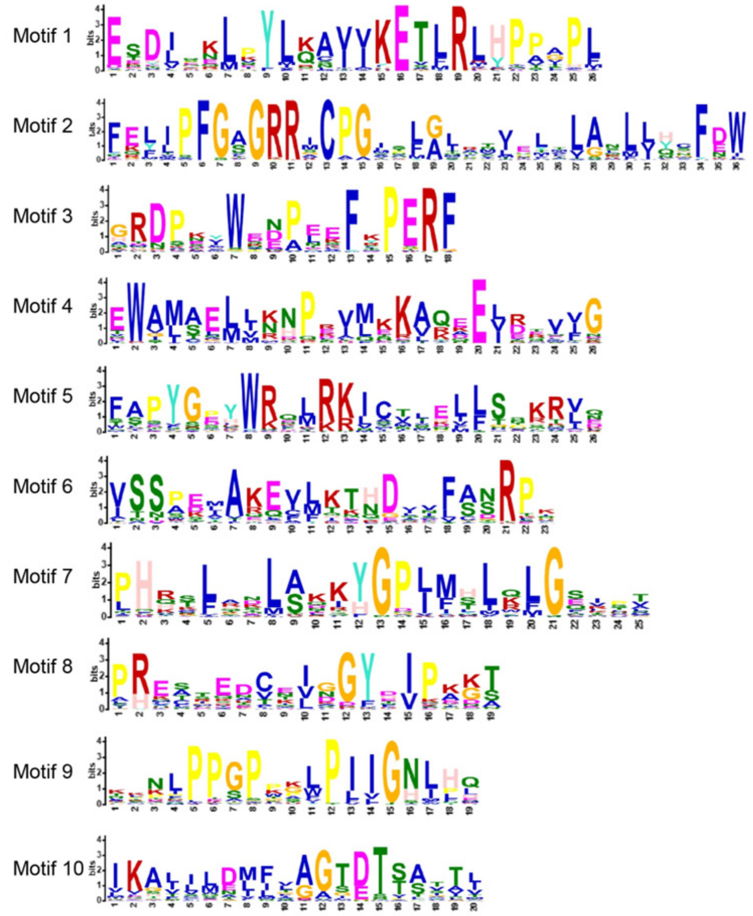

Figure S4. Dendrogram of all 24 samples from different growth stage of *A. mongholicus*.

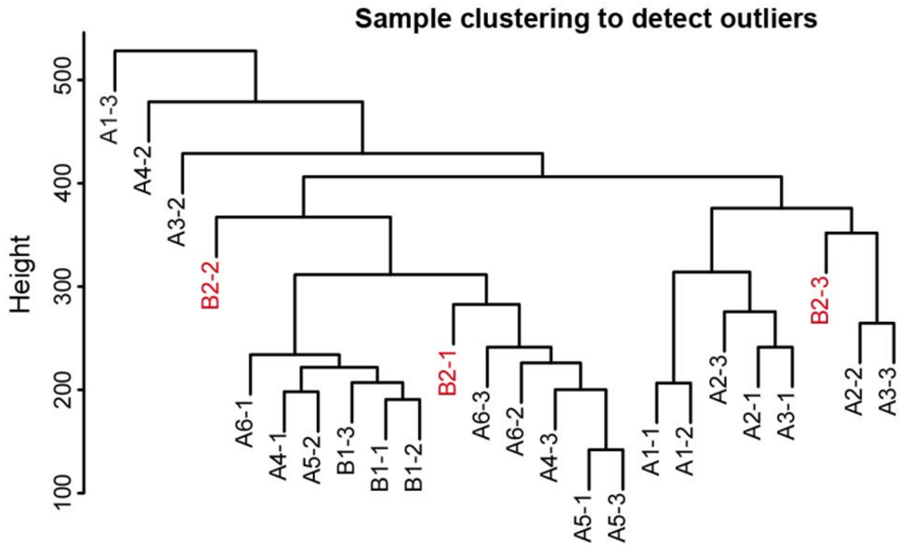

Figure S5. Relative expression of AmP450 genes in root, stem and leaf of 4-week-old *A. mongholicus* seedlings by qRT-PCR. (A) Blue module; (B) Grey module; (C) Other genes. The results are presented as the means ± standard error (n=3). Different letters indicate significant differences at $p < 0.05$, determined by Tukey's test of ANOVA.

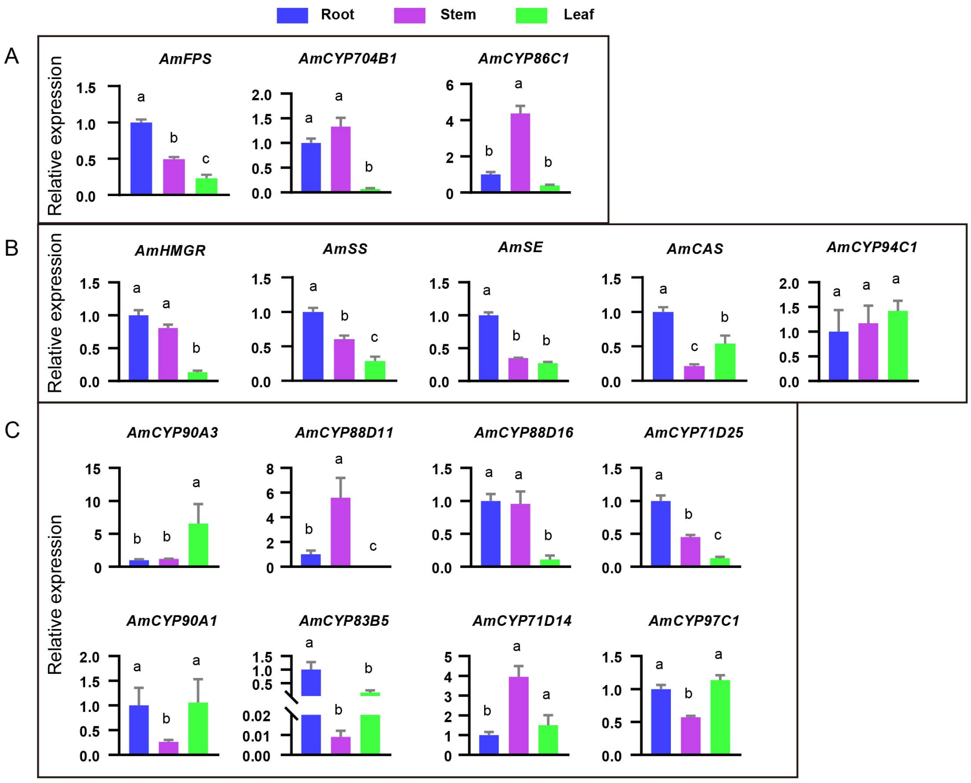

Supplement: Supplementary file 1 [file ijms-25-08333-s001.zip › Supplmental informations.pdf]
