# Supplementary material for: Identification and Expression Analysis of Cytochrome P450 Genes Probably Involved in Triterpenoid Saponins Biosynthesis in Astragalus mongholicus
Source: Int J Mol Sci. 2024 Jul 30;25(15):8333. doi: 10.3390/ijms25158333 (PMC11312233; doi:10.3390/ijms25158333)
Supplement: Supplementary file 1 [file ijms-25-08333-s001.zip › Table S1.pdf]

Table S1. Characterstics of P450 family members in *A. mongholicus*.

ID	CYP	coding sequence(bp)	amino acid	MW / KDa	PI	Localization*
Am01G000070.1	CYP736A14	1467	488	55.54	9.06	ER
Am01G000430.1	CYP704B1	1050	349	39.82	8.68	ER
Am01G000820.1	CYP71A25	1536	511	58.71	8.75	ER
Am01G000840.1	CYP71A28	1506	501	57.40	7.31	ER
Am01G001810.1	CYP90D1	1275	424	48.95	9.49	ER
Am01G002720.1	CYP88D12	1476	491	56.22	8.86	ER
Am01G002730.1	CYP88D15	1476	491	56.63	8.87	ER
Am01G002860.1	CYP82J4	1470	489	55.66	6.08	ER
Am01G002870.1	CYP82J5	1596	531	60.61	8.28	ER
Am01G003450.1	CYP81E3	1506	501	57.57	9.36	ER
Am01G004310.1	CYP714C1	1557	518	58.69	9.26	ER
Am01G005060.1	CYP51G1	1470	489	55.48	7.26	ER
Am01G005900.1	CYP78A3	1680	559	62.69	8.71	ER
Am01G008050.1	CYP86A8	1608	535	60.83	8.15	ER
Am01G009200.1	CYP704A3	1530	509	58.68	8.07	ER
Am01G009210.1	CYP704A2	1554	517	59.67	9.01	ER
Am01G009220.1	CYP704A1	1536	511	58.86	8.65	ER
Am01G009450.1	CYP705A12	1605	534	9.92	6.56	ER
Am01G011620.1	CYP76C2	1557	518	58.40	6.06	ER
Am01G011840.1	CYP72A154	1200	399	45.56	8.81	ER
Am01G013320.1	CYP703A2	1500	499	56.93	8.62	ER
Am01G018510.1	CYP707A2	1395	464	53.05	9.2	ER
Am01G020230.1	CYP84A3	1563	520	58.62	6.15	ER
Am01G020290.1	CYP90C1	1449	482	55.31	9.07	ER
Am01G020470.1	CYP722B1	1446	481	55.66	9.41	ER
Am01G020940.1	CYP90B2	1440	479	54.99	8.88	ER
Am01G022610.1	CYP81E1	1512	503	57.25	9.06	ER
Am01G025330.1	CYP706A1	1563	520	58.60	6.26	ER
Am01G026270.1	CYP71A27	1566	521	58.90	6.77	ER
Am01G026300.1	CYP71A7	1521	506	58.09	8.05	ER
Am01G029840.1	CYP734A1	1620	539	61.75	9.2	ER
Am01G038810.1	CYP74B29	1446	481	53.84	6.72	ER
Am02G002270.1	CYP74A1	1563	520	58.61	9.06	Chlo
Am02G002830.1	CYP707A1	1424	475	54.43	8.97	ER
Am02G004520.1	CYP711A1	1608	534	60.62	9.14	ER
Am02G008300.1	CYP83B5	1503	500	57.38	6.81	ER
Am02G009750.1	CYP721B1	1518	505	58.30	9.15	ER
Am02G010620.1	CYP88D14	1476	491	56.77	9.06	ER
Am02G011230.1	CYP74C1	1470	489	55.25	6.49	ER
Am02G011380.1	CYP88D6	1470	489	56.07	8.04	ER
Am02G011400.1	CYP71D26	1530	509	57.09	7.68	ER
Am02G011490.1	CYP88D7	1491	496	56.98	7.62	ER

Am02G011540.1	CYP71D15	1536	511	57.24	8.33	ER
Am02G011860.1	CYP96A1	1503	500	57.38	8.03	ER
Am02G012030.1	CYP76B3	1455	484	54.47	8.08	ER
Am02G012100.1	CYP86B1	1647	548	63.88	8.87	ER
Am02G012120.1	CYP707A4	1452	483	55.39	9.52	ER
Am02G017360.1	CYP94A1	1518	505	57.28	9.02	ER
Am02G018630.1	CYP97C1	1623	540	60.42	5.91	ER
Am02G021040.1	CYP93A6	1482	493	56.18	7.18	ER
Am02G021050.1	CYP93A4	1530	509	58.30	7.55	ER
Am02G021060.1	CYP93A2	1440	479	54.29	8.1	ER
Am02G021070.1	CYP93A1	1542	513	58.47	8.35	ER
Am02G030750.1	CYP76T25	1449	482	54.52	8.66	ER
Am02G030760.1	CYP76T24	1461	486	55.23	7.77	ER
Am02G030880.1	CYP77A3	1476	491	55.49	9.03	ER
Am02G031760.1	CYP76E1	1497	498	55.95	6.55	ER
Am02G034010.1	CYP715A2	1545	514	58.52	8.49	ER
Am02G035300.1	CYP76D1	1587	528	58.88	8.17	ER
Am02G038230.1	CYP73A100	1593	530	60.76	8.97	ER
Am02G040600.1	CYP76A1	1503	500	56.96	8.76	ER
Am03G002930.1	CYP722A1	1467	488	55.05	9.17	ER
Am03G003320.1	CYP74A2	1545	514	58.44	9.08	Chlo
Am03G006840.1	CYP711A2	1584	527	59.59	9.18	ER
Am03G011860.1	CYP714A2	1590	529	60.44	8.35	ER
Am03G011890.1	CYP714A1	1611	536	60.63	8.58	ER
Am03G016300.1	CYP71A29	1374	457	52.06	7.73	ER
Am03G016320.1	CYP71A30	2817	938	107.03	8.62	ER
Am03G016340.1	CYP71A3	1539	512	58.58	7.08	ER
Am03G016350.1	CYP71A6	1488	495	56.79	6.82	ER
Am03G018050.1	CYP94B4	1542	513	58.87	8.85	ER
Am03G019980.1	CYP78A8	1563	520	58.61	8.52	ER
Am03G022250.1	CYP90A3	1428	475	54.52	9.33	ER
Am03G024940.1	CYP73A99	1521	506	58.22	9.12	ER
Am03G025020.1	CYP79B2	1599	532	60.85	9.1	ER
Am03G025040.1	CYP728H1	1449	482	55.35	9.13	ER
Am03G026070.1	CYP71A4	1533	510	58.24	8.22	ER
Am03G026080.1	CYP71A5	1542	513	58.69	7.51	ER
Am03G026090.1	CYP79B3	1134	377	43.35	8.1	ER
Am03G026750.1	CYP86A1	1614	537	61.62	9.12	ER
Am03G028420.1	CYP71D10	1515	504	57.08	8.86	ER
Am03G028430.1	CYP71D9	1518	505	57.59	8.32	ER
Am03G028450.1	CYP71D13	1737	578	66.41	8.65	ER
Am03G028460.1	CYP71D11	1545	514	58.02	8.76	ER
Am03G028480.1	CYP71D12	1482	493	56.10	7.24	ER
Am03G028510.1	CYP71D22	1353	450	51.40	8.38	ER

Am03G031510.1	CYP75A3	1533	510	56.99	8.05	ER
Am03G031860.1	CYP75A1	1515	504	56.85	8.94	ER
Am03G033480.1	CYP75B1	1545	514	57.02	7.29	ER
Am03G036170.1	CYP727B1	1683	560	63.00	6.71	ER
Am04G002100.1	CYP97A1	1740	579	65.00	6.69	ER
Am04G002940.1	CYP87A3	1449	482	55.52	9.31	ER
Am04G004510.1	CYP88A85	1482	493	56.74	8.91	ER
Am04G005360.1	CYP81E8	1500	499	56.97	9.21	ER
Am04G005380.1	CYP81E2	1566	521	59.66	8.92	ER
Am04G006330.1	CYP71D8	1530	509	57.89	8.13	ER
Am04G006380.1	CYP71D7	1521	506	57.12	7.68	ER
Am04G006800.1	CYP90B1	1479	492	56.27	8.94	ER
Am04G007400.1	CYP90C2	1497	498	56.72	9.04	ER
Am04G007470.1	CYP84A1	1563	520	59.02	5.96	ER
Am04G009340.1	CYP707A3	1440	479	54.53	9.27	ER
Am04G009570.1	CYP89A1	1524	507	58.86	8.84	ER
Am04G015530.1	CYP78A4	1547	517	58.50	6.72	ER
Am04G016240.1	CYP79B1	1587	528	60.42	9.26	ER
Am04G020780.1	CYP86A2	1533	510	58.44	9.34	ER
Am04G021290.1	CYP83B4	1500	499	57.68	8.84	ER
Am04G021310.1	CYP83B6	1512	503	57.88	8.58	ER
Am04G022100.1	CYP73A98	1518	505	57.79	8.94	ER
Am04G024920.1	CYP90A1	1428	475	54.39	9.32	ER
Am04G025780.1	CYP701A16	1563	520	59.71	8.83	Chlo
Am04G028110.1	CYP718A1	1401	466	54.13	9.51	ER
Am04G028850.1	CYP728B1	1386	461	52.33	9.2	ER
Am04G029250.1	CYP71D21	1500	499	571.27	6.45	ER
Am04G029260.1	CYP71D19	1500	499	57.05	6.58	ER
Am04G029280.1	CYP71D14	1500	499	56.93	7.59	ER
Am04G029290.1	CYP71D18	1500	499	57.13	6.84	ER
Am04G029370.1	CYP71D20	1512	503	57.80	8.02	ER
Am04G029380.1	CYP71D23	1539	512	57.93	8.21	ER
Am05G000120.1	CYP76T26	1506	501	57.40	8.88	ER
Am05G000170.1	CYP94D2	1542	513	58.72	9.09	ER
Am05G001460.1	CYP94D3	1560	519	59.52	9.05	ER
Am05G004610.1	CYP88D10	1518	505	58.28	9.19	ER
Am05G004630.1	CYP71D17	1539	512	58.02	7.64	ER
Am05G004640.1	CYP88D13	1482	493	56.74	8.47	ER
Am05G004730.1	CYP71D24	1611	536	61.34	8.7	ER
Am05G004750.1	CYP88D16	1473	490	56.52	8.17	ER
Am05G004780.1	CYP71D16	1533	510	57.48	8.4	ER
Am05G004820.1	CYP71D25	1527	508	58.09	7.57	ER
Am05G004840.1	CYP88D11	1473	490	55.97	9.23	ER
Am05G008090.1	CYP72A15	1593	530	60.30	9.36	ER

Am05G010450.1	CYP89A2	1560	519	60.38	8.53	ER
Am05G010880.1	CYP76B1	1500	499	56.11	6.93	ER
Am05G010890.1	CYP76B2	1500	499	56.19	9.01	ER
Am05G011010.1	CYP76D2	1395	464	52.56	8.65	ER
Am05G012100.1	CYP86B2	1695	564	65.01	8.6	ER
Am05G013550.1	CYP84A2	1551	516	58.60	5.85	ER
Am05G018920.1	CYP94C1	1500	499	56.62	8.52	ER
Am05G018940.1	CYP94C2	1377	458	51.76	6.48	ER
Am05G022160.1	CYP72A153	1554	517	59.41	9.2	ER
Am05G023490.1	CYP89A3	1524	507	57.92	8.71	ER
Am05G024170.1	CYP707A5	1419	472	54.22	9.45	ER
Am05G025090.1	CYP93B17	1578	525	59.40	8.94	ER
Am05G025230.1	CYP82H2	1569	522	58.53	7.69	ER
Am05G025240.1	CYP82H1	1269	422	47.36	6.35	ER
Am05G025890.1	CYP71A26	1554	517	58.60	6.05	ER
Am05G028950.1	CYP716B1	1437	478	55.12	9.2	ER
Am05G030430.1	CYP81E6	1605	534	60.93	8.47	ER
Am05G030870.1	CYP78A6	1539	512	57.85	9.05	ER
Am05G032290.1	CYP81E5	1497	498	57.17	7.61	ER
Am05G032300.1	CYP81D2	1497	498	57.25	8.34	ER
Am05G032810.1	CYP716C2	1437	478	54.30	9.47	ER
Am05G033910.1	CYP716A1	1443	480	55.05	8.82	ER
Am05G035210.1	CYP86A3	1509	502	57.04	8.64	ER
Am05G037560.1	CYP736A12	1461	486	55.88	6.72	ER
Am05G038980.1	CYP736A13	2823	940	106.79	8.85	ER
Am05G038990.1	CYP736A15	1422	473	53.87	8.39	ER
Am06G010440.1	CYP88A4	1467	488	56.37	9.31	ER
Am06G010450.1	CYP88A3	1530	509	59.06	8.69	ER
Am06G012960.1	CYP93B16	1581	526	59.48	7.58	ER
Am06G012980.1	CYP81E4	1518	505	57.44	8.39	ER
Am06G013610.1	CYP72A65	1572	523	59.56	9.5	ER
Am06G013670.1	CYP72A64	1083	360	40.87	6.48	ER
Am06G017250.1	CYP82D47	1566	521	58.59	7.7	ER
Am06G018130.1	CYP734A2	1476	491	56.36	8.85	ER
Am06G021520.1	CYP72A66	1560	519	59.87	8.81	ER
Am06G021530.1	CYP72A70	1557	518	59.61	8.53	ER
Am06G021540.1	CYP72A337	1584	527	60.56	9.53	ER
Am06G021560.1	CYP72A68	1551	516	59.05	9.04	ER
Am06G025140.1	CYP77B1	1530	509	57.66	8.69	ER
Am07G000430.1	CYP96A2	1683	560	64.90	8.57	ER
Am07G001650.1	CYP82A3	1566	521	58.75	8.94	ER
Am07G001670.1	CYP82A2	1578	525	59.92	6.69	ER
Am07G001680.1	CYP82A4	1593	530	60.24	7.15	ER
Am07G001700.1	CYP82A6	1605	534	60.30	8.38	ER

Am07G001710.1	CYP82A5	1608	535	60.65	8.01	ER
Am07G001720.1	CYP82A9	1458	485	54.77	6.16	ER
Am07G003670.1	CYP85A1	1395	464	53.52	8.92	ER
Am07G004820.1	CYP735A1	1539	512	58.16	8.69	ER
Am07G007580.1	CYP82A1	1470	489	55.66	6.51	ER
Am07G008450.1	CYP733A5	1431	476	54.56	9.36	ER
Am07G008840.1	CYP72A69	1557	518	60.07	6.5	ER
Am07G008890.1	CYP88D8	1470	489	56.60	8.5	ER
Am07G008960.1	CYP72A71	1617	538	62.50	7.66	ER
Am07G008980.1	CYP72A72	1578	525	60.78	8.69	ER
Am07G009930.1	CYP715A3	1341	446	51.40	8.9	ER
Am07G009940.1	CYP715A1	1503	500	57.54	9.39	ER
Am07G012380.1	CYP720A1	1392	463	52.77	8.66	ER
Am07G013640.1	CYP88D9	1470	489	56.42	8.73	ER
Am07G015550.1	CYP83A1	1575	524	60.15	6.48	ER
Am08G000520.1	CYP735A2	1545	514	58.40	9.21	ER
Am08G001700.1	CYP77B2	1539	512	58.00	8.28	ER
Am08G003760.1	CYP94B3	1563	520	59.62	8.94	ER
Am08G005980.1	CYP724B1	1434	477	53.97	9.03	ER
Am08G007690.1	CYP714C2	1569	522	59.18	6.9	ER
Am08G008060.1	CYP93E2	1533	510	57.84	8.55	ER
Am09G003250.1	CYP714B1	1611	536	61.12	8.83	ER
Am09G003640.1	CYP76T27	1503	500	56.60	8.5	ER
Am09G003670.1	CYP76T28	1515	504	57.23	6.77	ER
Am09G005280.1	CYP97B1	1890	629	70.18	5.63	ER, Nucl
Am09G007040.1	CYP83B3	1536	512	58.04	6.22	ER
Am09G007050.1	CYP83B7	1461	486	55.28	7.91	ER
Am09G008050.1	CYP98A2	1464	487	56.18	9.25	ER
Am09G009400.1	CYP83A2	1557	518	59.58	6.53	ER
Am09G010880.1	CYP85A2	1530	509	57.68	7.25	ER
Am09G010920.1	CYP94D1	1506	501	57.73	8.63	ER
Am09G012660.1	CYP712C1	1629	542	60.42	8.2	ER
Am09G012670.1	CYP93A5	1479	492	56.08	6.33	ER
Am09G014390.1	CYP94A2	1524	507	57.93	9.39	ER
Amptg000100lG 000190.1	CYP71D27	1491	496	55.62	8.36	ER

MW: molecular weight; pI: theoretical isoelectric points;

* Chlo: Chlo; ER:Endoplasmic reticulum; nucl: Nuclear.
