# Supplementary material for: Identification and Expression Analysis of Cytochrome P450 Genes Probably Involved in Triterpenoid Saponins Biosynthesis in Astragalus mongholicus
Source: Int J Mol Sci. 2024 Jul 30;25(15):8333. doi: 10.3390/ijms25158333 (PMC11312233; doi:10.3390/ijms25158333)
Supplement: Supplementary file 1 [file ijms-25-08333-s001.zip › Table S4.pdf]

Table S4. Primers used in this study for qRT-PCR.

**Primers**	**Sequences (5'-3')**
18S-RT-F	TCAACCATAAACGATGCCGACC
18S-RT-R	TTTCAGCCTTGCGACCATACTCC
AmCYP71A28-RT-F	AGAGGCATGGAGTCAAAGCT
AmCYP71A28-RT-R	AGCTCCTCTGTTGCCTTCTT
AmCYP72A69-RT-F	ATGGGGATGGATGTTGCTGA
AmCYP72A69-RT-R	TGGGTTTGGATTTGGCTTCA
AmCYP51G1-RT-F	GGTGGAGGAGCAGAAGAAGT
AmCYP51G1-RT-R	GGTGGAGTCTCAAGGCTTCT
AmCYP76T28-RT-F	GCAGAGCTATTACGCAACCC
AmCYP76T28-RT-R	AATGGAGCTGGTGGGTGTAA
AmCYP78A3-RT-F	GCTGAAATCGCCTCCCAAAT
AmCYP78A3-RT-R	GTCCAAACACCGACCACATC
AmCYP88D8-RT-F	CACCTGAGACGTGCAAAAGA
AmCYP88D8-RT-R	ATGGGAGCAGTGACTAGACG
AmFPS-RT-F	CTGCTCATGGTGGGTGAGAA
AmFPS-RT-R	CACCCTGAAGATTGAGCTCGT
AmCYP98A2-RT-F	TTGACATGGCAGAGAACCCT
AmCYP98A2-RT-R	TATCAGCTGGCACACGTTTG
AmCYP87A3-RT-F	CCCCAGCTGTACATTTGAGC
AmCYP87A3-RT-R	CCCACCGAAAGCCATGAAAT
AmCYP704B1-RT-F	GTGATTGCTGGTCGAGACAC
AmCYP704B1-RT-R	ACACCCTCTTCCTTTGCTCT
AmCYP704A2-RT-F	CTGGCCCACGTATCTGCTTA
AmCYP704A2-RT-R	ACCTTGGAACTGCACAGAGA
AmCYP72A15-RT-F	GCGTTTGAAGGAACAAGGGT
AmCYP72A15-RT-R	AAAGGCAAAACACGAGGAGC
AmSS-RT-F	CTGCCAAAGTGATTGACCGA
AmSS-RT-R	TGAGCCATATGTGTTTGCACT
AmSE-RT-F	CTTGGAGGGATTCTGGCAAGT
AmSE-RT-R	TGTGTAGGCAAGGGCAGAAC
AmCAS-RT-F	TGGAATGTACCTCAGCAGCA
AmCAS-RT-R	ACTGCCCAAGAACCATACCA
AmCYP97A1-RT-F	TCCGCTATCTCAACAACCGT
AmCYP97A1-RT-R	CGCTTGGAGTGAGAATTGGG
AmCYP93A6-RT-F	TGCCAATGGTAGCGAGAGAA
AmCYP93A6-RT-R	CCTTCTTCCGGTTCCAAACG
AmCYP78A8-RT-F	ACTACTGGCTACTCCCTGGA
AmCYP78A8-RT-R	CAGCGAGTTTTCTATGGGCC
AmCYP72A72-RT-F	ATGGGGATGGATGTTGCTGA
AmCYP72A72-RT-R	ATCCGACAGTTCCATGGGTT
AmCYP74A1-RT-F	ACTGAACTCACTGGCGGTTA
AmCYP74A1-RT-R	AATGAACTCCGGGATGACGT

AmCYP72A153-RT-F	TCTTCACCCCTGTCCATCAC
AmCYP72A153-RT-R	TTTTGTGAAGTCCTGTGGCC
AmCYP93A5-RT-F	CCACACCAAGCACTTCACAA
AmCYP93A5-RT-R	AGCTGAACTATGAGGACGGT
AmCYP716B2-RT-F	ATATGCGCCACCACCCTATT
AmCYP716B2-RT-R	ACTGGCATTGGATCCCTACC
AmCYP86A3-RT-F	GGTTAGCCGAGCCATTAAGC
AmCYP86A3-RT-R	CTGACAGGGTTTGAGGGTCT
AmCYP94C1-RT-F	TCTTCTTGGCCGTGGAATCT
AmCYP94C1-RT-R	TTGAGGAAGCCATGAGAGGG
AmHMGR-RT-F	TGTTGAGAGTGTGACCGGAA
AmHMGR-RT-R	TGGAACCGTGTACTCCTTCC
AmCYP73A98-RT-F	CGCCATAATCGCAGTCACAA
AmCYP73A98-RT-R	GATCATCGCCGACTTGTAGC
AmCYP93B17-RT-F	TGTTGCCATGGTTCCCTTTG
AmCYP93B17-RT-R	GGTCCACTTGAGAAGCTCCT
AmCYP90A3-RT-F	GAGTCTCGGCTTACCCTTCA
AmCYP90A3-RT-R	GGTTCGTTTCAGGATCAGCC
AmCYP97C1-RT-F	AACAACACCACTCCTTTCGC
AmCYP97C1-RT-R	TGCCAGAATCGTCCTTTCCT
AmCYP76E1-RT-F	CGTTCGCCACCATGCTTAAT
AmCYP76E1-RT-R	GTTGACTCTCCCAGCATCCT
AmCYP88D7-RT-F	GAAAGCCAAGGAAGAGCAGG
AmCYP88D7-RT-R	CAGACGTTGCCTCTCGAAAG
AmCYP86B1-RT-F	GCAATGGGAAGAATGGAAAGC
AmCYP86B1-RT-R	TGTATATGATGCTGGCTGCAA
AmCYP93A1-RT-F	TTCACCCAACTGGGCCTTTA
AmCYP93A1-RT-R	CCATAGTGCTCTGTCCCTCT
AmCYP94A1-RT-F	CTCGGCAATGGCATCTTCAA
AmCYP94A1-RT-R	GAGGATTGGGAGAAGACGGT
AmCYP90A1-RT-F	AAACTACCACCGGGAACCTT
AmCYP90A1-RT-R	ACCGTTGGTTCACCAAACAC
AmCYP86A1-RT-F	TCTCCGAGTTGACGACAGTT
AmCYP86A1-RT-R	CCATCCGGCAATACATCGTC
AmCYP711A2-RT-F	GCAAAGCAATATGGCCCAATC
AmCYP711A2-RT-R	ATCTCGGGTGAAGAAGAGGC
AmCYP83B5-RT-F	CTTACCTCCTCGTCCACCTG
AmCYP83B5-RT-R	TCTTTGGCAATTTCGGCTGA
AmCYP71A30-RT-F	GGGTGCAATTCTTTCACTCCA
AmCYP71A30-RT-R	ACCAAAGCTACCACCACCAT
AmCYP71D14-RT-F	ACATCGTCAGGGATTGTGGT
AmCYP71D14-RT-R	GGATGCAGCCTCAGTGTTTC
AmCYP71D17-RT-F	TGGCCAAGGTGAAGAAGTAGTGGA
AmCYP71D17-RT-R	CCGATGTGTCACCGCCAGCA

AmCYP88D16-RT-F	GGGATGGCCTCTTCTTGGCA
AmCYP88D16-RT-R	ACATCTCAGGCTCGGACACA
AmCYP71D16-RT-F	TGTGGAGCTGGCTCTTGCAT
AmCYP71D16-RT-R	ACAAGGACGATAAGCTGTGGGT
AmCYP71D25-RT-F	TGTTGGTCGCCTTTTGCCAC
AmCYP71D25-RT-R	GGACCCTTGTTGTTGCTGCC
AmCYP88D11-RT-F	GAGGACAAGACCAGCCTCCC
AmCYP88D11-RT-R	CACGTCGGTTGTTGCCTCTC
AmCYP71D7-RT-F	GCCGCTGGAACTGATACATC
AmCYP71D7-RT-R	ACAAAGGAGAAGGTGGGTGT
